# The effect of cesarean delivery on the neonatal gut microbiome in an under-resourced population in the Bronx, NY, USA

**DOI:** 10.1186/s12887-024-04908-7

**Published:** 2024-07-13

**Authors:** Sandra E. Reznik, Ayodele J. Akinyemi, David Harary, Mariam S. Latuga, Mamta Fuloria, Maureen J. Charron

**Affiliations:** 1 Department of Pathology, Albert Einstein College of Medicine, Bronx, NY USA; 2 Department of Obstetrics and Gynecology and Women’s Health, Albert Einstein College of Medicine, 1300 Morris Park Avenue, Forch. 312, Bronx, NY 10461 USA; 3 Department of Pharmaceutical Sciences, St. John’s University, Queens, NY USA; 4 Department of Biochemistry, Albert Einstein College of Medicine, Bronx, NY USA; 5 Sidney Kimmel Medical College, Philadelphia, PA USA; 6 Department of Pediatrics, Division of Neonatology, The Children’s Hospital at Montefiore Albert Einstein College of Medicine, 1601 Tenbroeck Avenue, 2nd floor, Bronx, NY 10461 USA; 7 Department of Medicine & the Fleischer Institute for Diabetes & Metabolism, Albert Einstein College of Medicine, Bronx, NY USA

**Keywords:** Gut Microbiome, Under-resourced Population, Cesarean delivery, Pregnancy, Neonatal outcomes

## Abstract

**Background:**

Neonatal and early-life gut microbiome changes are associated with altered cardiometabolic and immune development. In this study, we explored Cesarean delivery effects on the gut microbiome in our high-risk, under-resourced Bronx, NY population.

**Results:**

Fecal samples from the Bronx MomBa Health Study (Bronx MomBa Health Study) were categorized by delivery mode (vaginal/Cesarean) and analyzed via 16 S rRNA gene sequencing at four timepoints over the first two years of life. Bacteroidota organisms, which have been linked to decreased risk for obesity and type 2 diabetes, were relatively reduced by Cesarean delivery, while Firmicutes organisms were increased. Organisms belonging to the *Enterococcus* genus, which have been tied to aberrant immune cell development, were relatively increased in the Cesarean delivery microbiomes.

**Conclusion:**

Due to their far-reaching impact on cardiometabolic and immune functions, Cesarean deliveries in high-risk patient populations should be carefully considered.

**Supplementary Information:**

The online version contains supplementary material available at 10.1186/s12887-024-04908-7.

## Background

Cesarean delivery, a surgical procedure involving the extraction of a fetus through the mother’s abdominal and uterine walls, has become increasingly prevalent worldwide [1]. While cesarean delivery is a life-saving intervention in certain medical scenarios, its rising rates, often attributed to non-medical factors, have raised concerns regarding potential impacts on neonatal health.

In the United States, approximately 32% of babies are born by Cesarean delivery [2]. Cesarean delivery has been linked to respiratory disorders, neurologic disorders (including autism spectrum disorder [3] and schizophrenia [4]), asthma [5], juvenile arthritis, celiac disease, type I diabetes mellitus and obesity [6, 7]. One area of particular interest is the neonatal gut microbiome, a complex ecosystem that plays a crucial role in immune system development, metabolism, and overall health [1, 8–10]. Gut dysbiosis at birth is associated with increased risk for metabolic disorders, including insulin resistance and Type I diabetes mellitus [6, 7]. In addition, abnormal gut microbiota have been linked to inflammatory disorders, including asthma, necrotizing enterocolitis and inflammatory bowel disease as well as neurodevelopmental disorders [3–5, 11, 12].

While the *in utero* environment was previously thought to be sterile, several recent studies have reported evidence of microbial DNA in the placenta [13], amniotic fluid [14] and meconium [15, 16], suggesting that the fetus is exposed to microorganisms before delivery. This line of reasoning implies that the inoculum acquired during vaginal delivery is not the neonate’s first exposure to microorganisms, and therefore may not be the primary factor in shaping the infant’s first microbiome community. If intrapartum inoculation with the mother’s commensural flora is not the most critical part of microbiome seeding, than mode of delivery, i.e. birth by Cesarean or vaginal delivery, may have less influence on the neonate’s microbiome.

In this study, we utilized the MomBa database to test our hypothesis that mode of delivery impacted the development of the gut microbiome at birth in a high-risk, under-resourced patient population. By focusing on this specific demographic, we aim to contribute valuable insights into the intersection of mode of delivery and the neonatal microbiome. Such knowledge is crucial for designing targeted interventions and improving maternal and neonatal healthcare practices, particularly in communities facing socioeconomic and health disparities.

## Methods

### Study population

The Bronx Mother Baby Health study (Bronx MomBa Health Study), an ongoing longitudinal study started in 2018, examines the pathogenesis of obesity in the first two years of life. The overall objective of the Bronx MomBa Health Study is to determine if an adverse developmental milieu associated with intrauterine growth restriction (IUGR) will alter DNA methylation profiles, resulting in functional changes in CD3^+^ T-cell subpopulations and subsequent childhood obesity at 24-months of age. A cohort of 411 mother-infant dyads who received prenatal care and delivered at a large urban private, non-profit hospital in the Bronx, New York, USA were screened for eligibility in the study and 163 were consented and enrolled. Inclusion criteria included healthy term appropriate for gestational age (AGA) and IUGR singleton infants born to mothers with no prenatal history of depression, smoking after the first trimester of pregnancy, or gestational/type 2 diabetes. Infants who were large for gestational age, and those with an Apgar score < 7 at 5 min of age, umbilical artery pH ≤ 7.25, chromosomal/congenital abnormalities, congenital infections, or inborn errors of metabolism were excluded. All infants enrolled in this study were admitted to the Newborn Nursery per the hospital protocol but roomed in with their mother unless they were receiving phototherapy or undergoing a procedure such as a circumcision. Infant anthropometric measurements, and cord- and peripheral blood samples were obtained at multiple timepoints during the first two years of life. Enrollment was paused temporarily between March 2020 through September 2020 during the COVID pandemic. The results presented here are from a subset of the larger Bronx MomBa Health Study cohort.

This study was approved by the Montefiore Medical Center/Albert Einstein College of Medicine Institutional Review Board (*NCT03402139*).

#### Sample collection

The Bronx MomBa Health study included 163 infants, and stool samples were obtained for one or more of the timepoints from a subset of subjects (87) who were included in this study. Stool samples were obtained at multiple timepoints during the first two years of life (at birth [within first three days of life], and at 6, 12 and 24 months of age). Stool samples collected at birth, while the newborn was still admitted in the Newborn Nursery, were either collected in a 2.0 mL microcentrifuge tubes (Cat No. NC9327032) from USA Scientific Inc and then frozen at -80^o^C (3.8% of samples at birth) or smeared on Whatman FTA™ Minicards (Cat No. WB120055) (96.2% of samples at birth) and then stored under vacuum at room temperature. Subsequent stool samples were collected at 6, 12 and 24 months of age during scheduled follow-up visits. While 6 frozen samples were collected initially, after quality control measures were performed only 1 frozen sample was acceptable and included in the study. For a small subset of patients, parents brought the most recent soiled diaper from less than 24 h prior to the scheduled study visit stored in a Ziploc bag with Tyvek^®^ silica gel desiccant packets at room temperature; during the visit, a small amount of the stool from the diaper was smeared on Whatman FTA™ Minicards. Some of the infants stooled during the study visit and the sample was collected soon thereafter. The samples were placed under vacuum conditions within 24 h of collection from the soiled diaper. Mostly, theparents collected the sample themselves on Whatman FTA™ Minicards soon after the infant stooled, placed the minicard in a biohazard bag with Tyvek^®^ silica gel desiccant packets and returned the sample to research personnel at the study visit or mailed ‘Priority Mail Express’ (USPS) by the parents to Albert Einstein College of Medicine, Bronx, NY where the samples were stored under vacuum at room temperature. We have determined feasibility of extracting DNA and 16S ribosomal RNA sequencing and amplification from these samples.

**Fig. 1:**
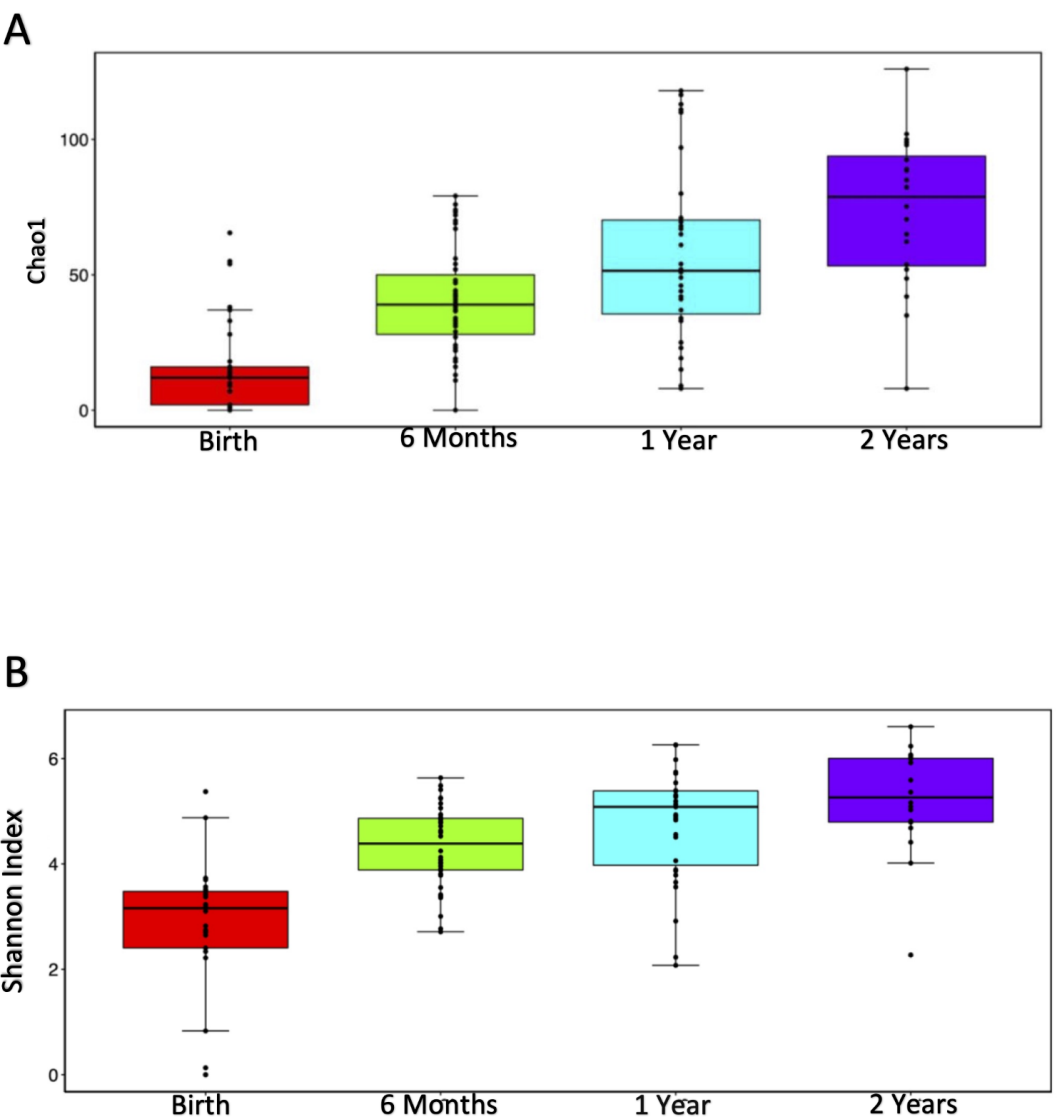
Gut microbiome diversity increases from birth to 2 years of age. 16 S ribosomal RNA sequencing and analysis were performed as described in the Methods. Chao1 (*P* = 2.85E-06, birth vs. 6 months; *P* = 0.032, 6 vs. 12 months; *P* = 0.029, 12 vs. 24 months, Panel A) and Shannon (*P* = 2.60E-07, birth vs. 6 months; *P* = 0.15, 6 vs. 12 months; *P* = 0.07, 12 vs. 24 months, Panel B) plots of alpha diversity of gut microbiome from birth to age 2. Statistical significance was determined by Kruskall Wallis testing

### DNA extraction and 16 S rRNA gene sequencing

Stool samples were sent to CD Genomics (Shirley, NY, USA) for gut microbiome analysis, as previously described [6]. CD Genomics performed the following procedures: genomic DNA extraction, polymerase chain reaction amplification of the V3-V4 region of 16 S rRNA gene using the primers 341 F = CCTACGGGNGGCWGCAG and 805R = GACTACHVGGGTATCTAATCC [17, 18] and purification and DNA library construction and sequencing. The overlapping regions between the paired-end reads were merged using FLASH v1.2.7 [19] and raw reads were quality filtered under specific filtering conditions to obtain high-quality clean tags on the basis of the QIIME2 [20] quality control process.

Sequences that were less than 200 bp in length or that contained homopolymers longer than 8 bp were removed. The chimera sequences were detected by comparing tags with the reference database (SILVA) [21] using the UCHIME [22] algorithm and then removed. The effective sequences were then used in the final analysis.

### Microbiome informatics and statistical analysis

QIIME2 (version: 2022.11.1) was used to cluster the tags with 97% similarity and acquire the operational taxonomic units (OTUs), then the OTUs were annotated based on SILVA taxonomic database (Max Planck Institute for Marine Microbiology and Jacobs University, Bremen, Germany). At the level of 97% similarity, we obtained the OTU number for each sample. The OTUs annotated as mitochondria, chloroplast, and unknown were removed. To assess species annotation resolution ratio of OTUs and species complexity for each sample, the statistical amounts of sequences of every sample in each classification level (Kingdom, Phylum, Class, Order, Family, Genus, and Species) were calculated. The abundance of information in each taxonomic level was generated with QIIME, and the microbial community structure graphs of each level were drawn by R language tool [23].

Alpha diversity indices (i.e., ACE, Chao1, Shannon, and Simpson) were calculated by QIIME2 from rarefied samples using richness and diversity indices of the bacterial community. Beta diversity was calculated using multiple algorithms including binary Jaccard, Bray-Curtis, and (un)weighted UniFrac (limited to bacteria), after which Intra-group and Inter-group beta distance boxplot diagrams were generated. A one-way analysis of similarity (ANOSIM) [24] as performed to determine the differences in bacterial communities among groups. Linear discriminant analysis (LDA) effect size (LefSe) analysis was performed to reveal the significant ranking of abundant modules in all samples [25]. A size-effect threshold of 4.0 on the logarithmic LDA score was used for discriminative functional biomarkers. Phylogenetic Investigation of Communities by Reconstruction of Unobserved States (PICRUSt) [26] was used to predict the functional gene content in the fecal microbiota based on taxonomy obtained from the Greengenes reference database1 [27]. PICRUSt and LefSe were performed online in the Galaxy workflow framework. Through the corresponding green gene identification of each OTU, we obtained the related Kyoto Encyclopedia of Genes and Genomes (KEGG) and Cluster of Orthologous Group (COG) family information, the abundance of these KEGG and COG families was calculated.

G-test in Statistical Analysis of Metagenomic Profiles (STAMP) [28] was used for large sample sizes (annotated functional gene number > 20) and Fisher test was used for small sample sizes (annotated functional gene number < 20) for the species abundance at the genus level to test for significance. Adjusted P values, sometimes called Q values, take the number of tests into account, and are more stringent than non-adjusted P values. Finally, analysis of composition of microbiomes (ANCOM) [29] was done to test for significant differences between microbial populations. Chao and Shannon results were analyzed by Kruskall Wallis testing.

## Results

### Patient demographics and mode of delivery

Table 1 compares demographic characteristics in patients with vaginal vs. Cesarean deliveries at all four timepoints. No significant differences in maternal age, pre-pregnancy BMI, prevalence of antibiotic use, prevalence of hypertensive disorders of pregnancy, infant sex, gestational age, neonatal birthweight or neonatal intrauterine growth restriction status were observed across all groups. Overall, 41% (15/37) percent of vaginal deliveries were induced using either misoprostol or cervical foley.

**Table 1:** Patient demographics

	Birth (*N* = 26)		6 months (*N* = 35)		12 months (*N* = 30)		24 months (*N* = 20)	
	Vaginal Delivery (*N* = 19)	C-section (*N* = 7)	Vaginal Delivery (*N* = 12)	C-section (*N* = 23)	Vaginal Delivery (*N* = 12)	C-section (*N* = 18)	Vaginal Delivery (*N* = 5)	C-section (*N* = 15)
Maternal age (years; mean ± SD)	27.84 ± 6.2	30.14 ± 5.08	29.75 ± 4.56	32.43 ± 6.14	27.75 ± 5.88	34.28 ± 4.80	29.40 ± 4.51	31.93 ± 3.95
Race (N; %)								
White Black Others	7 (36.8) 8 (42.1) 4 (21.1)	3 (42.9) 1 (14.3) 3 (42.9)	10 (83.3) 1 (8.3) 1 (8.3)	14 (60.9) 8 (34.8) 1 (4.3)	8 (66.7) 3 (25.1) 1 (8.3)	9 (50.0) 8 (44.4) 1 (5.6)	3 (60.0) 2 (40.0) 0 (0.0)	7 (46.7) 8 (53.3) 0 (0.0)
Ethnicity (N; %)								
Hispanic Non-Hispanic	14 (73.7) 5 (26.3)	6 (85.7) 1 (14.3)	10 (83.3) 2 (16.7)	14 (60.9) 9 (39.1)	10 (83.3) 2 (16.7)	12 (66.7) 6 (33.3)	4 (80.0) 1 (20.0)	7 (46.7) 8 (53.3)
Pre-pregnancy BMI (mean ± SD)	29.65 ± 5.28	30.98 ± 4.72	28.83 ± 3.51	30.26 ± 7.16	28.70 ± 5.08	32.13 ± 5.64	29.04 ± 4.40	30.43 ± 6.32
Received intrapartum antibiotics (N; %)	12 (63.2)	7 (100.0)	6 (50.0)	22 (95.7)	4 (33.3)	18 (100.0)	2 (40.0)	14 (93.3)
Hypertensive disorders present during pregnancy (N; %)	2 (10.5)	1 (14.3)	0 (0.0)	6 (26.1)	2 (16.7)	4 (22.2)	0 (0.0)	2 (13.3)
Infant sex (N; %)								
Male Female	8 (42.1) 11 (57.9)	3 (42.9) 4 (57.1)	5 (41.7) 7 (58.3)	10 (43.5) 13 (56.5)	7 (58.3) 5 (41.7)	11 (61.1) 7 (38.9)	3 (60.0) 2 (40.0)	9 (60.0) 6 (40.0)
Gestational age (weeks; mean ± SD)	39.42 ± 1.09	38.79 ± 1.4	39.59 ± 0.99	39.00 ± 0.87	39.78 ± 1.05	39.20 ± 1.14	39.63 ± 0.72	38.69 ± 0.79
Birthweight (Kg; mean ± SD)	3.22 ± 0.55	3.26 ± 0.46	3.04 ± 0.61	3.24 ± 0.43	3.10 ± 0.55	3.33 ± 0.42	3.12 ± 0.57	3.31 ± 0.35
IUGR status (N; %)	4 (21.1)	1 (14.3)	6 (50.0)	3 (13.0)	5 (41.7)	1 (5.6)	2 (40.0)	1 (6.7)

### Evolution of the gut microbiome from birth to age two

**Fig. 2:**
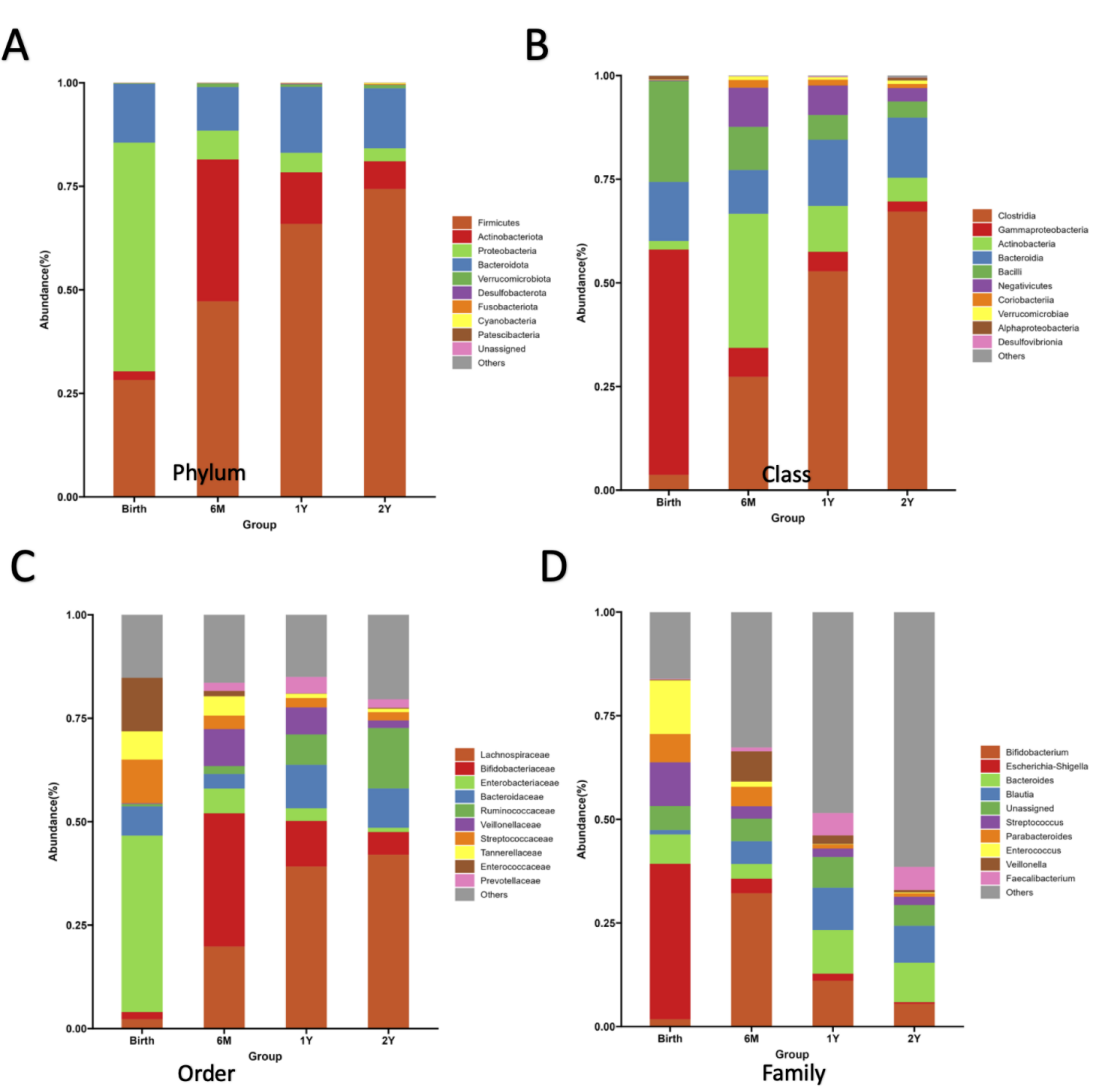
Gut microbial communities shift dramatically from birth to 2 years of age. Abundance histograms showing beta diversity between different timepoints from birth to age 2. Each bar represents the total sum of the microorganisms in all the fecal samples collected at one timepoint (birth, 6 months, 1 year or 2 years, as indicated). Each color represents a taxonomy, and the length of each color block represents the proportion of the relative abundance of the taxonomy. Four different taxonomic classification levels are shown: phylum (**A**), class (**B**), order (**C**) and family (**D**). Statistically significant differences in the abundance of specific organisms at different timepoints were found by ANCOM, as described in Results section

Over the first two years of life, as expected, there was a notable increase in gut microbiome diversity among our study participants, which is reflected by the increase in alpha diversity as depicted in Chao1 and Shannon plots (Fig. 1A and B, respectively). Differences in alpha diversity between the various timepoints were statistically significant. By Chao, *P* = 2.85E-06, birth vs. 6 months; *P* = 0.032, 6 vs. 12 months; *P* = 0.029, 12 vs. 24 months (Fig. 1A). By Shannon, *P* = 2.60E-07, birth vs. 6 months; *P* = 0.15, 6 vs. 12 months; *P* = 0.07, 12 vs. 24 months (Fig. 1B). Abundance histograms of the babies’ fecal microbiota from birth to age two revealed distinct shifts in microbial communities. At six months of age, a major shift occurred at the phylum level, with Firmicutes and Actinobacteriota increased compared to Proteobacteria (6 Month Group compared to Birth Group, Fig. 2A). The Firmicutes population continued to expand from 6 months to 2 years, while Actinobacteriota levels peaked at 6 months. ANCOM revealed that the changes in Firmicutes and Actinobacteriota levels were statistically significant (W = 12 and 13, respectively). In addition Bacteroidota increased at 1 year of age (W = 11). As Firmicutes increased relative to Proteobacteria at the phylum level, at the class level, Clostridia, Actinobacteria and Negativicutes microbiota, which are rare in the neonatal microbiome, were detected in almost all fecal samples collected at six months or later (Fig. 2B). These differences were statistically significant by ANCOM (W = 20, 17 and 18, respectively). The decline in organisms in the Bacilli class after birth was also significant (W = 17). Finally, the changes in Coriobacteriia and Desulfovibrionia were significant as well (W = 16 and 15, respectively). Further, at the order level, Lachnospiraceae and Bifidobacteriaceae microbiota, which were rare at birth, began to appear at six months of age with Lachnospirales being common at ages 1 and 2 (Fig. 2C). By ANCOM, these changes in abundance of Lachnospiraeae and Bifidobacteriaceae organisms were significant (W = 48 and 43, respectively). At the family level, Escherichia-Shigella and Enterococcus were prevalent at birth, but decreased by 6 months, although this change was not significant by ANCOM. Bifidobacterium appeared with a relative peak at 6 months (Fig. 2D, W = 87 by ANCOM). Unweighted and weighted principal coordinate analysis (PCoA) (Supplemental Fig. S1A and S1B, respectively), which tests for beta diversity, confirms that gut microbial populations in the babies in our study changed significantly over time, with microbiota at birth showing separation from microbiota at six months (adjusted P values, unweighted and weighted PCoA’s, 0.0015 and 0.003, respectively) and six months showing distinct separation from microbial populations seen at 1 year (adjusted P values, unweighted and weighted PCoA’s, 0.03 and 0.09, respectively).

**Fig. 3:**
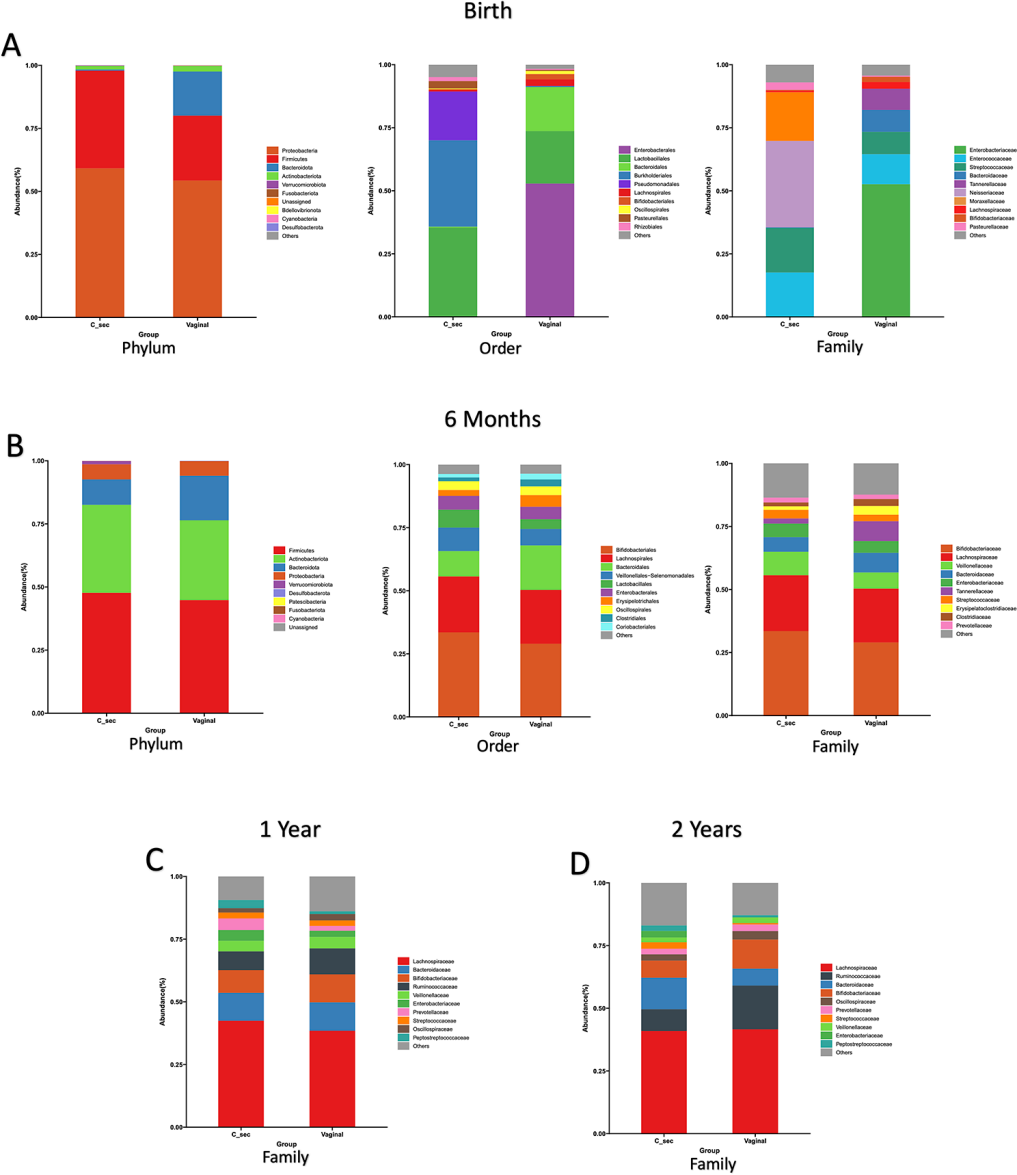
Mode of delivery affects the composition of gut microbial communities at birth. Abundance histograms showing beta diversity between samples collected from neonates born by vaginal vs. Cesarean delivery. Each bar represents the total sum of the microorganisms in all the fecal samples collected from neonates who were born by either mode of delivery at one timepoint as shown: birth (**A**), 6 months (**B**), 1 year (**C**) or 2 years (**D**). Three different taxonomic classification levels (phylum, order, family) are shown for birth and 6 months; one taxonomic level (family) is shown for 1 year and 2 years. Increases in organisms in the Burkholderiales order and in the Neisseriacea family in Cesarean deliveries were statistically significant (W = 14 and 86, respectively, by ANCOM, Panel **A**.) No significant differences were found at 6, 12, or 24 months (Panels **B-D**)

### Effect of cesarean delivery on fecal microbiome composition from birth to two years

Abundance histograms reveal differences in the gut microbial communities at birth between babies born via vaginal delivery vs. Cesarean delivery at all taxonomic levels. At the phylum level, Bacteroidota organisms make up a larger portion of the gut microbiome in babies delivered vaginally, but not in babies delivered by Cesarean section (Fig. 3). Cesarean section neonates, on the other hand, have a greater abundance of Firmicutes organisms, which appear to have replaced the Bacteroidota. At the order level, Enterobacterales and Bacteroidales make up increased portions of the gut microbiome in the neonates delivered vaginally but are absent in the microbiota of the babies delivered by Cesarean section. Lactobacillales is increased in the Cesarean section neonates. In addition, two orders of microorganisms, Burkholderiales and Pseuomonadales, are not present in vaginally delivered babies but are abundant in neonates delivered by Cesarean section (Fig. 3). At the family level, Enterobacteriaceae are abundant in vaginally delivered neonates but absent in babies born by Cesarean delivery. Nesseriaceae and Moraxellaceae, on the other hand, appear only in the neonates delivered by Cesarean section. The disparity in the gut microbiomes seen at birth is greatly reduced by the age of six months and is not seen at one or two years of age (Fig. 3). The statistical significance of these differences was determined by ANCOM. At birth, the increases in organisms in the Burkholderiales order and in the Neisseriacea family in Cesarean deliveries were significant (W = 14 and 86, respectively). None of the major components of the microbiota at 6, 12 or 24 months was significantly different between the vaginal and Cesarean delivery groups.

**Fig. 4:**
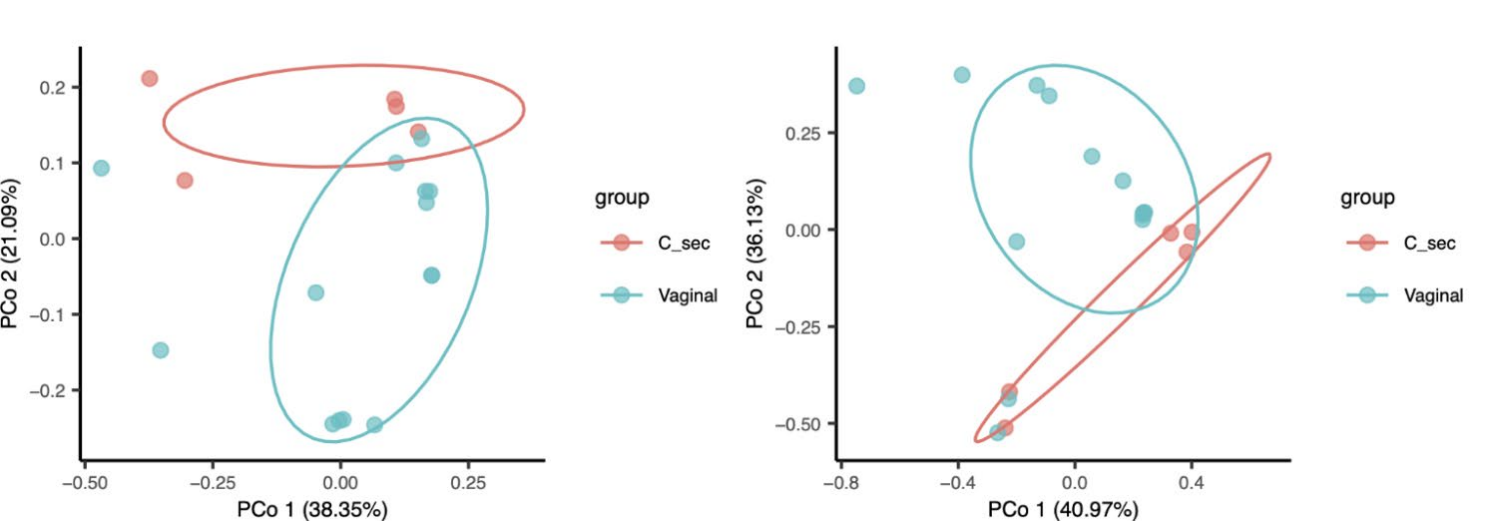
PCoA shows partial separation of the gut microbial communities based on mode of delivery. Unweighted (**A**) and weighted (**B**) PCoA of gut microbiome based on vaginal vs. Cesarean delivery

**Fig. 5:**
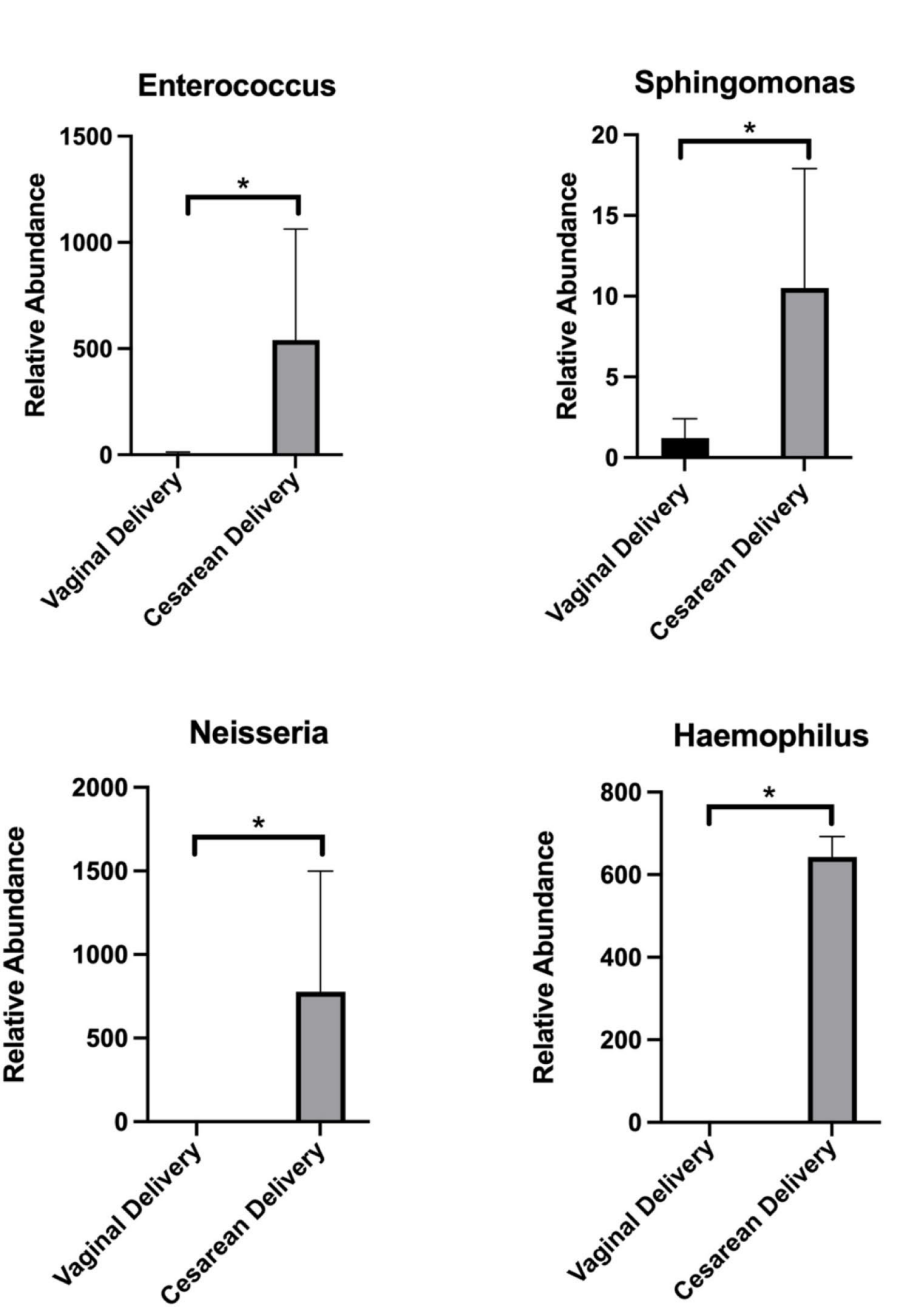
Significantly increased gut micro-organisms associated with Cesarean delivery. Relative abundance of organisms belonging to the *Enterococcus* (**A**), *Sphingomonas* (**B**), *Neisseria* (**C**) and *Haemophilus* genuses. Neisseria and Haemophilus organisms were only detected in fecal samples from Cesarean deliveries. *P < 0.05

Unweighted and weighted PCoA provides a visual display of the separation between the microbiomes between the two groups (vaginal vs. Cesarean delivery) at birth (Fig. 4). Although the PCoA did not reveal a statistically significant separation between the two groups, we tested for differences in relative abundance of specific microorganisms at the genus level. Four genuses were increased in relative abundance in Cesarean delivered compared to vaginally delivered microbiomes at birth: *Enterococcus* (541.8 *±* 522.1 vs. 7.1 *±* 5.4, *P* = 0.049*)*; *Sphingomonas (*10.5 *±* 7.4 vs. 1.2 *±* 1.2, *P* = 0.043); *Neisseria* (777.5 *±* 721.5 vs. 0 *±* 0, *P* = 0.039) and *Haemophilus* (64.3 *±* 49 vs. 0 *±* 0, *P* = 0.014) (Fig. 5). Interestingly, no genuses were increased in relative abundance in Cesarean deliveries at 6, 12, or 24 months, but several were decreased in fecal samples from babies born by Cesarean section compared to vaginally delivered babies including *Collinsella* at 6 months; *Akkermansia*, *Megasphaera*, *Enterococcus*, *Lactobacillus* and *Olsenella* at 12 months; and *Subdoligranulum*, *Fusicatanibacter*, *Lachnospira*, *Paraprevotella*, *Lachnospiraceae*, *UCG.004*, *Sutterella* and *Holdemanella* at 24 months.

**Fig. 6:**
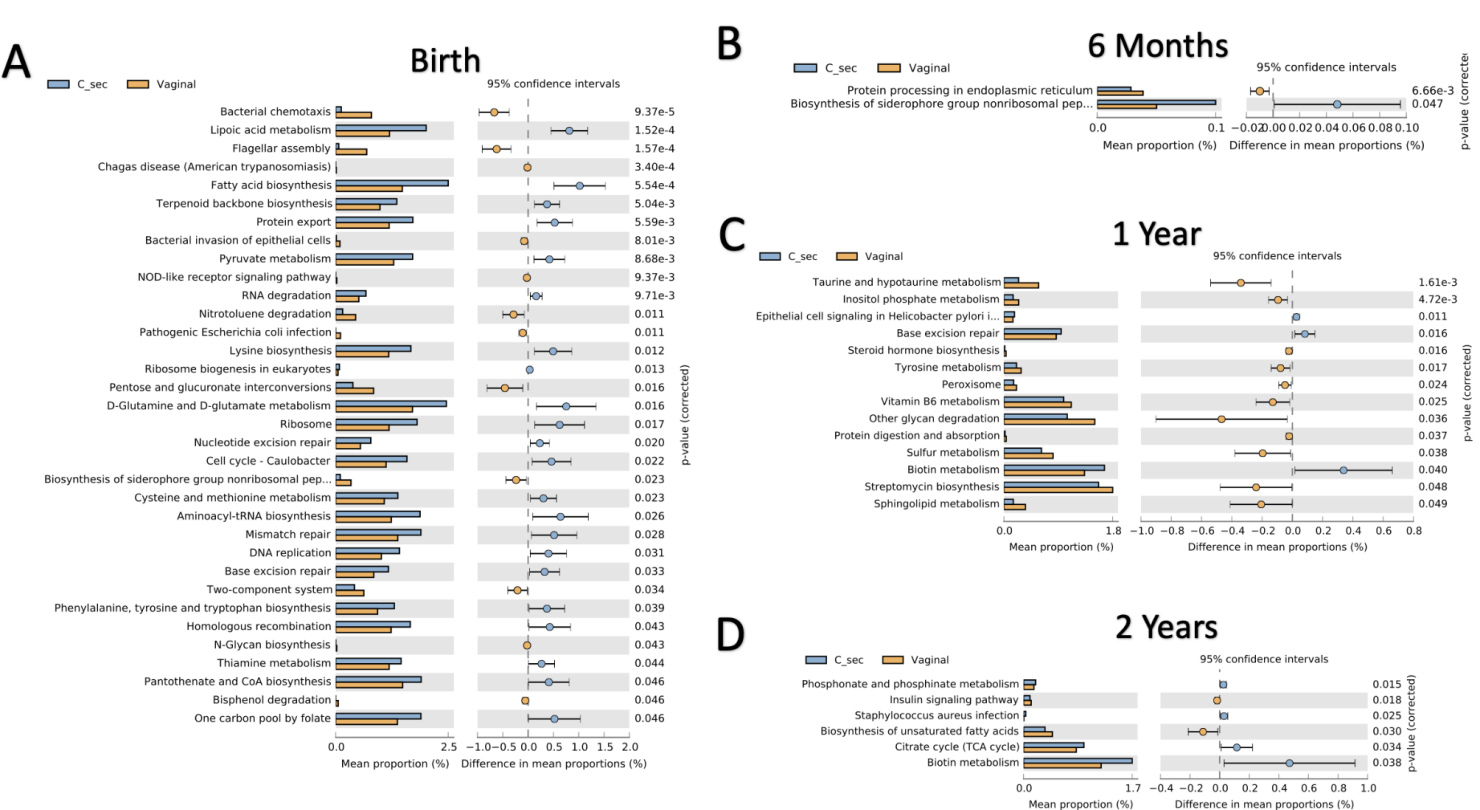
KEGG analysis of Cesarean vs. vaginally delivered microbiomes. Significantly differentially expressed pathways are shown at birth (**A**), 6 months (**B**), 1 year (**C**) and 2 years (**D**)

### Effect of cesarean delivery on fecal microbiome biological pathways from birth to two years

Using KEGG pathway analysis, we compared the predicted activity levels of biological pathways in the gut microbiomes collected from the two groups, vaginal vs. Cesarean deliveries (Fig. 6). At birth, a total of 34 pathways were predicted to be differentially expressed, with 22 pathways up-regulated in Cesarean subjects but only 12 pathways up-regulated in vaginal births (Fig. 6A). Many of the pathways increased in the Cesarean subjects were related to metabolism, e.g. pyruvate metabolism, fatty acid biosynthesis, lysine biosynthesis, tryptophan biosynthesis and lipoic acid synthesis. We consulted the KEGG database and compared the metabolism related pathways that were up-regulated in our Cesarean delivery samples to metabolic pathways that are relatively up-regulated in *Enterococcus*, *Sphinogomonas*, *Neisseria* and *Haemophilus* organisms (the four types of organisms increased in our Cesarean delivery fecal samples). All the pathways mentioned above are up-regulated in all four genuses. In addition, terpenoid biosynthesis is up-regulated specifically in *Enterococcus* and *Neisseria*.

Besides metabolic pathways, DNA synthesis and repair pathways were up-regulated in Cesarean delivery gut microbiota at birth, including DNA replication, base excision repair, mismatch repair and homologous recombination pathways (Fig. 6A). In contrast, the most up-regulated pathways in vaginal delivery subjects at birth included bacterial motility pathways, such as bacterial chemotaxis and flagellar assembly. Many fewer pathways were differentially expressed later in infancy. Moreover, at age 1 year, almost all the up-regulated metabolic pathways were now associated with vaginal delivery, a reversal of the trend observed at birth.

## Discussion

The worldwide rate of Cesarean sections has increased from approximately 7% in 1990 to 21% today, with 32.1% of neonates being born by Cesarean section in the United States in 2022 [2]. The increase in Cesarean section rate is in part related to patient preferences rather than medical indications [30]. At our institution, the Cesarean section rate is approximately 37% which is also indicative of the greater disease burden in our pregnant mothers; approximately three-fourths of the pregnant mothers seen within our delivery system are either overweight/obesity and 40% have hypertensive disorders of pregnancy. At the same time, being born by Cesarean section increases lifelong susceptibility to non-communicable diseases and immune disorders [30]. Here we report differences in the gut microbiome between vaginally and Cesarean delivered neonates at birth.

In general, in our patient population, at birth, the composition of the gut microbiome in vaginally delivered neonates was more closely related to the vaginal commensal flora predicted to be present in their mothers, while the gut microbiomes of babies delivered by Cesarean section more closely resembled skin flora and hospital microbial communities, similar to what has been reported in other patient cohorts [33, 34]. Notably, at the phylum level, Proteobacteria comprised a relatively higher proportion of the microbiome in vaginal delivery cases compared to Cesarean delivery cases. Proteobacteria consume oxygen and lower redox potential and are believed to play a role in preparing the gut for the strict anaerobes that inhabit the intestine following the initial period around birth [35]. Loss of this critical group of organisms could therefore compromise the development of a normal gut microbiome. Furthermore, Cesarean delivered neonates had relatively lower amounts of *Bacteroides* species at birth, which aligns with previous reports on the effect of mode of delivery on the neonatal microbiome [36, 37]. *Bacteroides* organisms and their short chain fatty acid metabolites such as butyric acid and propionic acid, induce peripheral Tregs in vitro and in vivo [38, 39]. In addition, low levels of *Bacteroides* organisms at birth has been linked to the development of atopic dermatitis [40] and food allergies [41]. At the order level, Burkholderiales, a soil and water micro-organism that began to colonize healthcare facilities in the 1970s (with the advent of newer antibiotics) [31] was only present in Cesarean delivery cases. At the genus level, *Sphingomonas* organisms, which are not part of normal human flora but have been found in hospital intensive care units [31] were only present in Cesarean delivery samples.

Some of the changes in the microbiome composition associated with mode of delivery in our study may be linked to increased disease susceptibility. For example, in our data set, infants born by Cesarean delivery had prominent *Enterococcus faecalis* and associated lipoic acid metabolism. In adult models of gut dysbiosis, *Enterococcus* has been associated with a pro-inflammatory state [42]. Specifically, stool from patients with ulcerative colitis and Crohn’s disease had higher abundance of *E.faecalis* compared to healthy controls. When stool samples from patients with inflammatory bowel disease were transplanted into germ free mice, lipoic acid metabolism in the germ free mice was associated with a probiotic cluster of *E.faecalis* strains [43]. It is possible that a relative abundance of *Enterococcus* in the developing microbiome may lead to an inflammatory mileu and negatively impact immune development. This hypothesis is supported by evidence from studies in germ free mice that the neonatal gut microbiome affects the development of subsequent immune function [38, 44, 45]. Moreover, intestinal bacteria and their short chain fatty acid metabolites have been linked to the development of regulatory T cell, helper T cell and B cell function [46]. The combination of reduced *Bacteroides* and increased *Enterococcus* species has been likened to a “stunted” microbiome allowing for growth of opportunistic organisms [47].

Importantly for our high-risk patient population, levels of *Bacteroides* species are positively correlated with leanness in mice [48] and inversely correlated with risk for type 2 diabetes mellitus [49]. Hence, the loss of *Bacteroides* species in the Cesarean delivery samples in our patient cohort, from which we excluded mothers with gestational or type 2 diabetes, supports the hypothesis that Cesarean delivery is a risk factor for metabolic dysfunction. Several mechanisms linking neonatal gut dysbiosis with increased weight gain have been proposed. These include altered gene expression in the host resulting in a shift toward a more calorie conserving transcriptome; direct effects on extraction of nutrients from food in the intestine, known as increased energy harvest; and increase in systemic inflammation mediated by release of lipopolysaccharide [49, 50].

Differences in the gut microbiome related to mode of delivery have been previously reported to affect development of neonatal immune and metabolic biological pathways [30, 49]. These effects are apparent in the KEGG analysis in our study where many metabolic pathways were up-regulated at birth in babies delivered by C-section, including lipoic acid metabolism, fatty acid biosynthesis and terpenoid backbone biosynthesis, suggesting a dysregulation of metabolic function that may manifest later in life in the form of cardiometabolic disease. On the other hand, pathways related to cell motility, i.e., flagellar assembly and bacterial chemotaxis, were up-regulated in vaginal deliveries. Exposure to these bacterial pathways may initiate the development of anti-bacterial immune responses in the neonate. Taken together, the KEGG results suggest that the gut microbiome in vaginally delivered neonates, as compared to Cesarean delivered infants, stimulates the development of neonatal metabolic and immune systems.

Similar to previous reports, we found that the effects of the mode of delivery which were evident at birth on abundance histograms rapidly changed by six months. Hence, effects of the altered microbiome composition may persist long after the shifts in microbial populations have disappeared. Moreover, samples collected after six months will be affected by several exogenous factors that have not yet come into play at birth, e.g. breastmilk vs. formula feeding, initiation of solid feeding, antibiotic use, early childhood infections, etc.

Our maternal population had higher rates of comorbidities such as chronic hypertension and overweight/obesity which may limit the generalizability of the data to a general population of well pregnant persons. Despite this limitation, our findings in our high risk MomBa patient cohort are consistent with the growing body of literature on the effects of mode of delivery on the neonatal microbiome. Therefore, Cesarean deliveries for non-medical reasons in high risk patients should be very carefully considered due to their potential long-term impact on the neonatal microbiome. In instances where a Cesarean delivery cannot be avoided, a deeper understanding of the biology of the neonatal microbiome may lead to postpartum interventions, e.g. treatment with probiotics, to counter some of the unfavorable effects of the altered gut flora.

### Electronic supplementary material

Below is the link to the electronic supplementary material.

Supplementary Material 1

## Data Availability

The raw microbiome data have been archived in the NCBI Sequence Read Archive (SRA) repository under the accession number PRJNA1123264.
