# Supplementary figures and images for: The effect of cesarean delivery on the neonatal gut microbiome in an under-resourced population in the Bronx, NY, USA
Source: BMC Pediatr. 2024 Jul 13;24:450. doi: 10.1186/s12887-024-04908-7 (PMC11245842; doi:10.1186/s12887-024-04908-7)

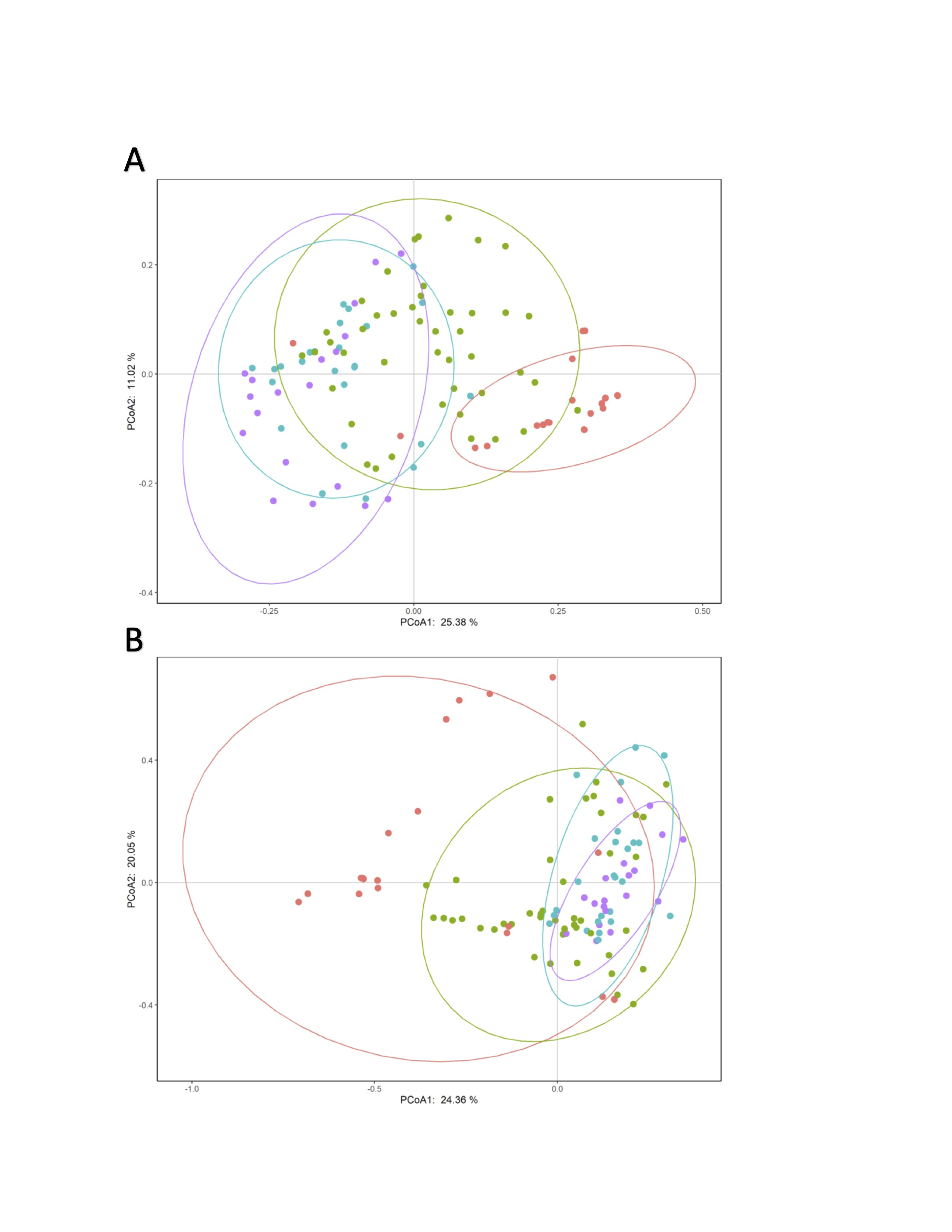

Supplement: Supplementary file 1 — Supplementary Material 1 [file 12887_2024_4908_MOESM1_ESM.tiff]
